# The impact of antibiotic use on transmission of resistant bacteria in hospitals: Insights from an agent-based model

**DOI:** 10.1371/journal.pone.0197111

**Published:** 2018-05-14

**Authors:** Jonatan Almagor, Elizabeth Temkin, Itzhak Benenson, Noga Fallach, Yehuda Carmeli, on behalf of the DRIVE-AB consortium

**Affiliations:** 1 Laboratory of Geosimulation and Spatial Analysis, Department of Geography and Human Environment, Tel Aviv University, Tel Aviv, Israel; 2 Department of Epidemiology and Preventive Medicine, Tel Aviv Sourasky Medical Center, Tel Aviv, Israel; 3 Sackler Faculty of Medicine, Tel Aviv University, Tel Aviv, Israel; Purdue University, UNITED STATES

## Abstract

Extensive antibiotic use over the years has led to the emergence and spread of antibiotic resistant bacteria (ARB). Antibiotic resistance poses a major threat to public health since for many infections antibiotic treatment is no longer effective. Hospitals are focal points for ARB spread. Antibiotic use in hospitals exerts selective pressure, accelerating the spread of ARB. We used an agent-based model to explore the impact of antibiotics on the transmission dynamics and to examine the potential of stewardship interventions in limiting ARB spread in a hospital. Agents in the model consist of patients and health care workers (HCW). The transmission of ARB occurs through contacts between patients and HCW and between adjacent patients. In the model, antibiotic use affects the risk of transmission by increasing the vulnerability of susceptible patients and the contagiousness of colonized patients who are treated with antibiotics. The model shows that increasing the proportion of patients receiving antibiotics increases the rate of acquisition non-linearly. The effect of antibiotics on the spread of resistance depends on characteristics of the antibiotic agent and the density of antibiotic use. Antibiotic's impact on the spread increases when the bacterial strain is more transmissible, and decreases as resistance prevalence rises. The individual risk for acquiring ARB increases in parallel with antibiotic density both for patients treated and not treated with antibiotics. Antibiotic treatment in the hospital setting plays an important role in determining the spread of resistance. Interventions to limit antibiotic use have the potential to reduce the spread of resistance, mainly by choosing an agent with a favorable profile in terms of its impact on patient's vulnerability and contagiousness. Methods to measure these impacts of antibiotics should be developed, standardized, and incorporated into drug development programs and approval packages.

## Introduction

Antibiotics have drastically reduced deaths and complications caused by bacterial infections and set the stage for modern medicine [1, 2]. However, the wide use of antibiotics has a price. Antibiotic use promotes the emergence and spread of resistant strains, leading to the exhaustion of this limited resource. After 70 years of excessive antibiotic use by humans, the World Health Organization warns that the increase in antibiotic-resistant bacteria (ARB) "threatens the achievements of modern medicine" [3]. Some predict that soon antibiotic resistance will lead to more deaths than cancer [4]. Humans can acquire ARB in two ways: occasionally, by *de novo* emergence of a mechanism of resistance in an individual patient, or, much more commonly, by transmission of bacteria that are already resistant. Some resistance mechanisms (e.g., VanA, CTX-M extended-spectrum beta-lactamases, KPC and NDM carbapenamases) arose in human pathogens only in a few instances during evolution, but have become widespread through transmission [5, 6]. Highly transmissible successful bacterial clones (such as *E*. *faecium* CC 17, *E*. *coli* ST131 or *K*. *pneumoniae* clonal group 258) that carry these mechanisms of resistance often drives their wide dissemination. Thus, it is transmission that turns rare events into a major public health problem [7].

For many ARB hospitals are the focal points for spread, with transmission occurring from patient to patient via contact with contaminated healthcare workers (HCW), other patients, or objects. The transmission dynamics of ARB in hospitals is therefore influenced by the interactions of patients and HCW and their specific characteristics and behavior [8–10]. In hospitals, transmission of ARB is accelerated by antibiotic pressure [11–13]. Several observational or quasi-experimental studies of interventions to reduce the use of certain types of antibiotics in hospitals reported a subsequent decline in ARB [14–16]; one modeling study found that limiting antibiotic use could decrease or increase ARB, depending on the antibiotic class [17].

Antibiotic treatment may facilitate the transmission of ARB in two ways. First, antibiotics disrupt the commensal flora that protect against colonization by invading bacteria, increasing patients' vulnerability to acquire new strains [18]. Thus, patients who are exposed to ARB during or shortly after antibiotic treatment are at a higher risk of becoming colonized [19–21]. Studies have reported a 2-9-fold increased risk of ARB acquisition among patients treated with various antibiotics; this increased risk differs between antibiotic agents [22–24]. Second, in patients who are already colonized by ARB, antibiotics eradicate competing commensal organisms that are antibiotic-sensitive, allowing overgrowth of those that are resistant. The increased load of ARB leads to enhanced shedding and thus to greater contagiousness [19, 23]. Studies have measured a 3.5–5.3 fold greater risk for ARB contamination in the immediate surroundings of colonized patients who received antibiotics, and have linked antibiotic use with colonized patients who were heavy dispersers [24, 25]. Furthermore, colonized patients not treated with antibiotics were found to have an 80% lower risk of room contamination [26].

The proportion of patients treated with antibiotics in hospitals varies greatly, between 21% and 55% according to European reports [27]. Even greater variation (up to 22-fold) is found when examining specific antibiotic agents used in different wards housing patients with similar characteristics [28]. This high variability suggests that the use of antibiotics is amendable.

Here, we used an agent-based model (ABM) to investigate the impact of antibiotic treatment on the transmission dynamics of ARB in a hospital, and the potential of antibiotic stewardship interventions in limiting the spread. The ABM enables us to answer questions where controlled experimentation is complex and may not be feasible [29–31]. We address the overall effect of antibiotic pressure on the spread of resistance under various conditions of antibiotic density, antibiotic agents' characteristics, prevalence level of ARB, and the contagiousness of the ARB strain.

## Materials and methods

### Model overview

The model is implemented in GAMA 1.7 modeling environment [http://gama-platform.org/]. The setting of the model is a typical hospital (Table 1). Agents consist of patients and health care workers (HCW). Contacts between patients and HCW and between adjacent patients are explicitly simulated; these contacts may result in transmission of ARB. Patients admitted to the hospital are assigned to an available bed in one of the wards and their length of stay (LOS) is determined based on a random draw from a lognormal distribution. A fraction of admitted patients is already colonized; since ARB carriage is correlated with an extended length of stay, colonized patients are assigned with LOS from a lognormal distribution with a higher average [32–34] (Table 1). In addition, acquisition of ARB during hospital stay prolongs the patient's stay. In accordance with studies on the average duration of colonization we assume that patients remain colonized for the whole duration of their stay [34–38]. On admission, a fraction of patients are prescribed antibiotic treatment. We define two types of antibiotic effect on treated patient: V-type is the increase in the vulnerability of a susceptible patient to acquire ARB and represents the fold increase in the risk of acquisition; C-type is the increase in the contagiousness of a colonized patient and represents the fold increase in the risk of the patient to transmit ARB. The duration of the treatment is assigned for 10 days or until the patient is discharged (in case patient's LOS is less than10 days). We consider the prescription of antibiotics as independent of the colonization state of the patient; that is, the antibiotics are prescribed to treat or prevent infections caused by other pathogens. Ward staff are assigned to specific patients within a single ward: each ward staff member is responsible for 8 patients within the ward ("staff cohorting"). General hospital staff members are not affiliated with a single ward; they can have contact with patients in any of the wards. Each type of HCW is characterized by a contact frequency with patients and can be transiently contaminated with ARB during a contact with a colonized patient. ARB contamination is cleared if infection control measures such as hand hygiene are implemented by HCW before or after contact with a patient.

**Table 1:** Hospital parameters used in the simulations.

**Parameter**	**Value**	**Note**
Hospital		
Capacity	800 beds	
Number of wards	20	40 patients per ward
HCW		
Ward staff	5 per ward	
General hospital staff	80	Moves across wards
Ward staff contact with patients	4 per hour	Assigned to 8 patients
General hospital staff contact with patients	3 per hour	
Compliance with standard infection control	50% of opportunities	Hand hygiene opportunities are before and after contact with a patient
Patients		
Average LOS, susceptible	6 days	Lognormal distribution
Average LOS, colonized	12 days	Lognormal distribution
Extension of LOS after acquiring ARB	50%	
Percent colonized on admission	1% or 10%	

### Transmission of ARB and the impact of antibiotic treatment

ARB transmission is defined by three basic probabilities: a) β_P_—probability of susceptible patients to acquire ARB during contact with a contaminated HCW; b) β_H_—probability of HCW contamination during contact with a colonized patient; c) α- probability per day of susceptible patients to acquire ARB from an adjacent colonized patient. In the model, antibiotic use increases the risk of transmission. V and C represent the fold increase in the risk of ARB acquisition (for susceptible patient) or of transmission (for colonized patient), respectively, during antibiotic treatment. When antibiotic is not used by the patient then V = 1 and C = 1. The following equations determine the probability of transmission between agents:

Probability for susceptible patient to acquire ARB (P_p_by_p_) from an adjacent colonized patient, per day:

Pp_by_p=α*V*C (1)

Probability for susceptible patient to acquire ARB (P_p_by_h_) during a contact with a contaminated HCW:

Pp_by_h=βP*V (2)

Probability for HCW to be contaminated (P_h_by_p_) during a contact with a colonized patient:

Ph_by_p=βH*C (3)

### Simulation experiments

Transmission probabilities are based on values estimated in the literature. Since the estimated values in those studies include the impact of antibiotics (the study population includes a mix of patients treated and not treated with antibiotics), we selected values slightly lower than what was found in the literature as the baseline probabilities for patients who are not treated with antibiotics. (Table 2). Patient-to-patient transmission probability (α) is based on studies that estimated acquisition risk in patients whose prior room occupant was a carrier of ARB. Using simulations, we examine the influence of 4 different variables on transmission of ARB:

1. Characteristics of the antibiotic agent: the fold increase in the risk of ARB acquisition (V) or transmission (C) during antibiotic treatment; these values vary in simulations between 1 and 4. The tested values are within the range of increased risk for acquisition observed among patients treated with various antibiotics [23–25], and the increased risk for ARB shedding and contamination of the surroundings by colonized patients who received antibiotics [20, 24–26].
2. Antibiotic density: the prevalence of antibiotic use is varied by changing either the proportion of admitted patients which are treated with antibiotics (0–100%) or the duration of antibiotic treatment (7 or 10 days).
3. ARB prevalence among admitted patients: an early stage of ARB spread in which 1% of admitted patients are carriers, and an endemic stage in which 10% are carriers.
4. Transmissibility of the ARB: a less transmissible strain (ARB-L) and a highly transmissible strain (ARB-H) (Table 2).

**Table 2:** Baseline transmission probabilities, by ARB strain.

Transmission probabilities	ARB-L	ARB-H	Values cited in the literature	Source
β_H_−Contamination of a HCW from a colonized patient, per contact	5%	10%	6%-40%	[39–44]
β_P_−Transmission from a contaminated HCW to a susceptible patient, per contact	2.5%	5%	6%-20%	[23, 39–41]
α–Transmission from a colonized patient to a susceptible adjacent patient, per day	0.5%	1%	1%-3%	[45–47]

For each scenario, the average of 100 simulations is presented as the results.

## Results

### The effect of antibiotic characteristics

Antibiotic agents may differ in their effects on patients' vulnerability (V) and contagiousness (C). The effect of these antibiotic characteristics on the transmission of ARB was examined. Transmission of ARB-L and ARB-H was simulated for various combinations of V and C. In these simulations 1% of admitted patients carry the ARB strain and 40% of admitted patients receive antibiotics.

**Fig 1:**
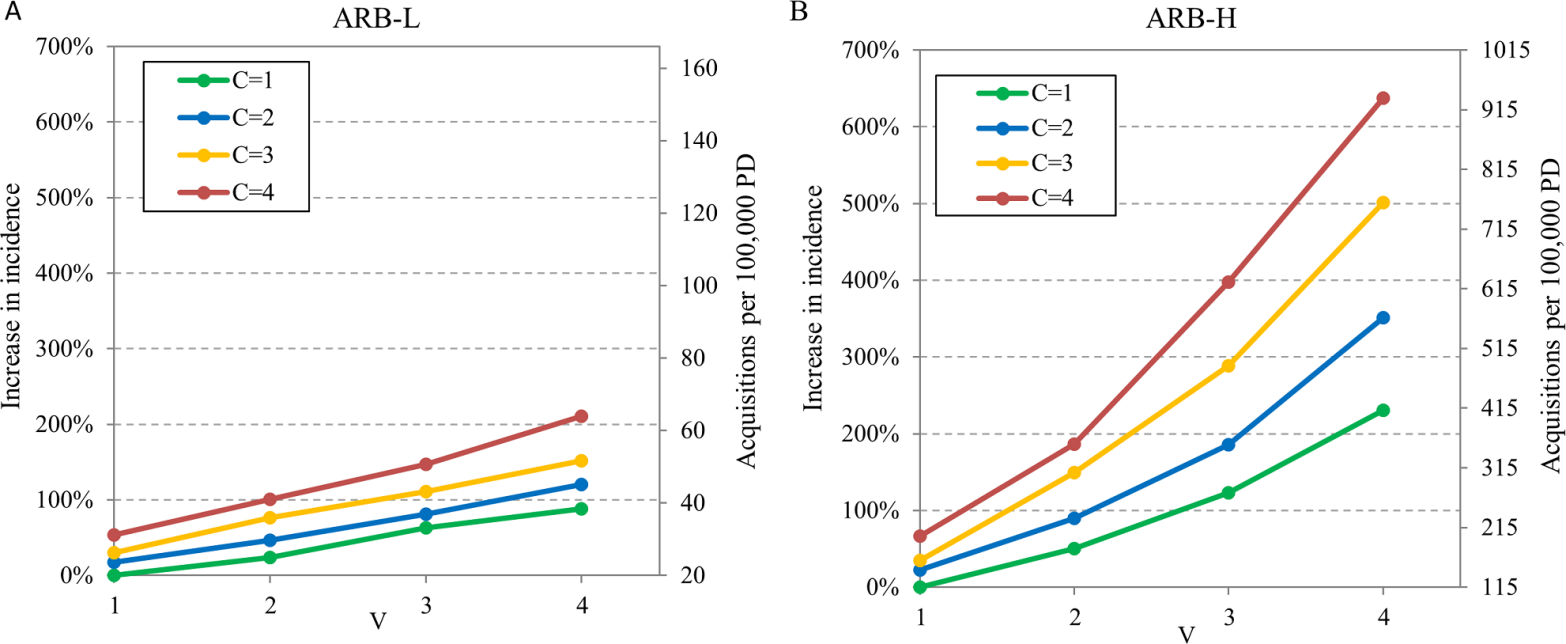
Impact of antibiotic characteristics on acquisition of ARB. Increase in incidence of acquisition (left axis) of (A) strain ARB-L and (B) strain ARB-H relative to no antibiotic impact, and ARB acquisitions per 100,000 patient days (PD) (right axis) for various values of an antibiotic's impact on vulnerability (V) of susceptible patients and on contagiousness (C) of colonized patients. In the simulated scenario 40% of admissions receive antibiotics and 1% of patients are colonized on admission. Note that when V = 1 and C = 1, antibiotics have no effect on acquisition, or, alternatively, no patients are receiving antibiotics.

Under conditions of no antibiotic pressure (C = 1 and V = 1, the antibiotic agent does not change contagiousness or vulnerability), the acquisition incidence for the ARB-L strain is 20 per 100,000 patient-days (PD) (Fig 1A, right axis, green line at V = 1). When an antibiotic agent that increases either V or C is used, the acquisition rate increases; a synergistic effect on the incidence of ARB acquisition is seen when an antibiotic agent that increases both V and C is used. For example, when an agent that increases both vulnerability and contagiousness of the treated patient by four folds (V = 4, C = 4) is used, the incidence rate increases by 230% (from 20 to 66 acquisitions per 100,000 PD) (Fig 1A). For the more transmissible strain ARB-H, the baseline acquisition incidence with no antibiotic pressure is much higher: 115 acquisitions per 100,000 PD (Fig 1B, right axis, green line at V = 1). When an antibiotic increases vulnerability and contagiousness by four folds (V = 4, C = 4) is used, the incidence rate increases by 679% and reaches 896 acquisitions per 100,000 PD (Fig 1B). Thus, antibiotic treatment has a greater impact on transmission when the ARB strain is more transmissible. Furthermore, as the impact of the antibiotic increases, ARB transmission increases non-linearly, approaching exponential increase for a highly transmissible strain.

We can also infer that an antibiotic's effect on increasing the vulnerability of susceptible patients has a greater influence on acquisition dynamics than its effect on increasing the contagiousness of colonized patients. Fig 1B depicts this difference in the case of ARB-H. When using an antibiotic agent that does not increase vulnerability (V = 1) but increases contagiousness by 2–4 fold (2 ≤ C ≤ 4), the acquisition rate relative to no antibiotic effect increases by 23% - 67% (moving vertically from the blue to the red line in Fig 1B). When using an antibiotic agent that does not increase contagiousness (C = 1) but increases vulnerability by 2–4 fold (2 ≤ V ≤ 4), the acquisition rate relative to no antibiotic effect increases by 50% - 230% (green line in Fig 1B). This is also true, but to a lesser degree, in the case of the less transmissible ARB strain (Fig 1A).

### The impact of antibiotic density on incidence of ARB acquisition

**Fig 2:**
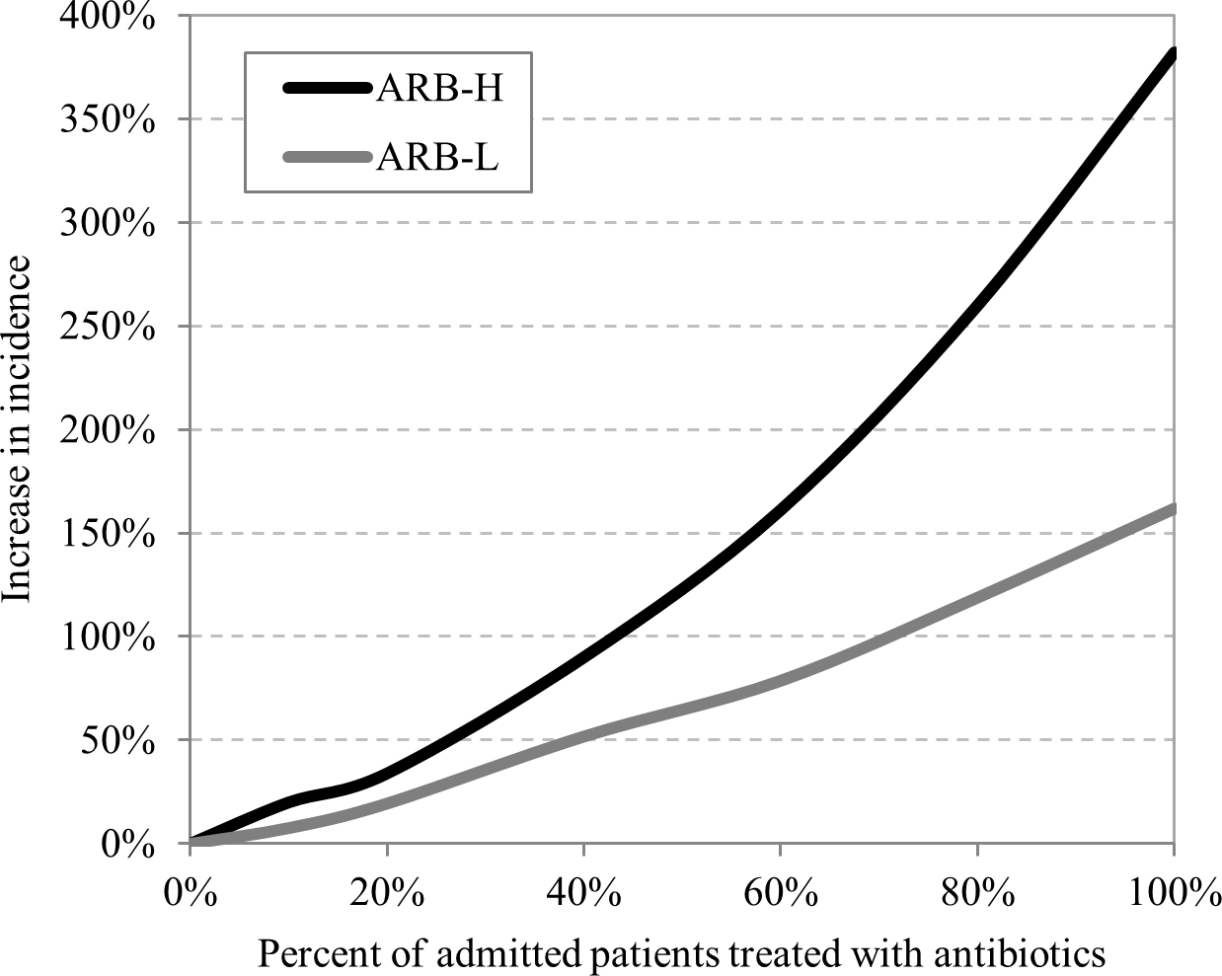
Increase in ARB acquisitions relative to no antibiotic use as antibiotic density increases. The ARB prevalence on admission is 1%, and antibiotic impact is V = 2, C = 2. With no antibiotic use, the number of ARB acquisitions per 100,000 patient-days is 20 for ARB-L and 115 for ARB-H. The % increase in incidence (y axis) refers to these baseline rates.

We varied antibiotic density by changing the proportion of admitted patients prescribed antibiotics for 10 days, and explored the effect on the incidence of ARB-L and ARB-H acquisition. In these simulations we modeled an antibiotic that doubles both vulnerability and contagiousness (V = 2, C = 2), and an early stage of ARB spread (1% are carriers upon admission). The simulations demonstrated that an increase in antibiotic density leads to a non-linear increase in the incidence of ARB acquisition, which approaches exponential increase in the case of ARB-H (Fig 2). When antibiotic density is high, the slope of ARB acquisition is steeper, indicating that reductions in antibiotic use will have a greater impact on ARB transmission in settings with high antibiotic density. Comparing the two resistant strains shows that the impact of antibiotic treatment on the transmission rate is greater for ARB-H than for ARB-L. This suggests that the spread of highly transmissible ARB is much more sensitive to changes in antibiotic density, particularly in high antibiotic density conditions.

### The impact of antibiotic density on the individual risk for ARB acquisition

To examine the significance of ARB prevalence on transmission dynamics, we modeled an antibiotic agent that doubles vulnerability and contagiousness (V = 2, C = 2). We varied antibiotic density by changing the proportion of admitted patients treated with antibiotics. Simulations included both early and established stages of ARB spread (1% vs. 10% carriers upon admission).

**Fig 3:**
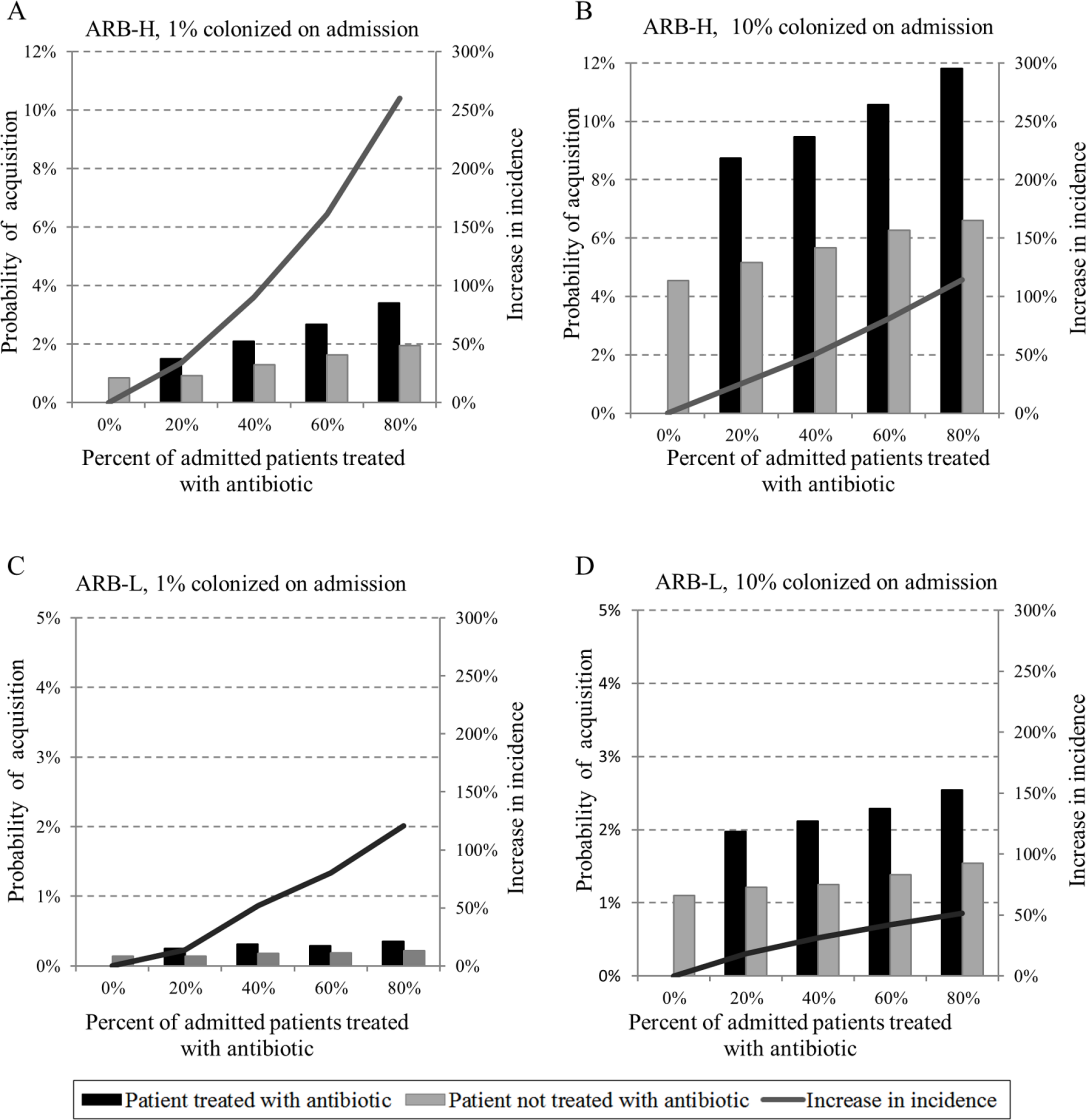
Individual risk for ARB acquisition. Probability of ARB-H and ARB-L acquisition during a 6-day hospital stay for patients treated and not treated with antibiotics (left axis) and the increase in incidence relative to the scenario without antibiotic use (right axis); (A) 1% of admissions are colonized with ARB-H (B) 10% of admissions are colonized with ARB-H (C) 1% of admissions are colonized with ARB-L (D) 10% of admissions are colonized with ARB-L. Antibiotic agent impact: V = 2, C = 2.

First, we examined how increasing antibiotic density affects the incidence of acquisition of ARB-H and ABR-L compared to a baseline transmission with no antibiotic use (Fig 3, solid line, right axis). In both stages of ARB spread an increase in antibiotic density leads to an increase in ARB acquisitions. However, in the stage where of ARB spread is established, the impact of antibiotic density on ARB transmission is lower. For instance, in the early stage of spread at high antibiotic density (80% of admitted patients are treated) incidence increase by 260% (Fig 3A) compared to 114% in an established stage of the spread (Fig 3B). This elevated impact in early stage of the spread is more moderate in case of ARB-L (Fig 3C and 3D).

Increasing antibiotic density affects an individual patient's risk of acquiring ARB. We measured the risk as the probability of acquisition during a typical hospital stay of 6 days. The probability of acquisition for a patient not treated with antibiotics is calculated as: the number of patients not treated with antibiotics who acquired ARB divided by the total number of patients not treated with antibiotics.

The probability of acquisition for a patient treated with antibiotics is calculated as: the number of patients treated with antibiotics who acquired ARB divided by the total number of patients treated with antibiotics. Since the average antibiotic treatment time was 4.5 days, we adjusted the probability to account for 6 days with antibiotic treatment.

The individual risk increases in parallel to the proportion of patients treated with antibiotics (Fig 3, bars, left axis). This is true both for patients treated and not treated with antibiotics. In the scenario of an early stage of spread, the resulting risk to an individual patient of acquiring ARB-H is 0.85% when no patients receive antibiotics (Fig 3A). When 20% of admitted patients are treated with antibiotics, the risk for a patient who is treated with antibiotics nearly doubles to 1.5% (Fig 3B, black bar). The risk increases 4-fold (to 3.4%) when 80% of admitted patients receive antibiotics, and doubles to 1.95% for patients not treated (Fig 3A). In an established stage of ARB spread, the risk for a patient treated with antibiotics doubles to 8% when 20% of patients admitted receive antibiotics, and nearly triples to 11% when 80% of admissions receive antibiotics. In an established stage of ARB spread, the effect of antibiotic density on the acquisitions is more modest; in an-early stage, increasing antibiotic density from 20% to 40% increases the risk of acquisition by 40% compared to only 20% in an established stage. Similar effects on an individual's risk for acquisition is also evident for ARB-L, but to a lesser degree (Fig 3C and 3D).

### Antibiotic stewardship interventions

Last, we explored the potential effect of antibiotic stewardship interventions recommended by the Infectious Diseases Society of America (IDSA) and the Society for Healthcare Epidemiology *o*f America (SHEA) in order to limit resistance [48]. We examined interventions that decrease the number of patients treated with antibiotics, shorten the duration of antibiotic courses, and replace antibiotic agents with high impact values (V and C) with ones with low impact. The simulated scenarios included 1% and 10% prevalence of ARB-H colonization on admission. ARB-H acquisitions following the simulated intervention were compared to a reference scenario in which 40% of admissions receive antibiotic agent A with pronounced effects on vulnerability and contagiousness (V = 4, C = 4) for 10 days.

**Fig 4:**
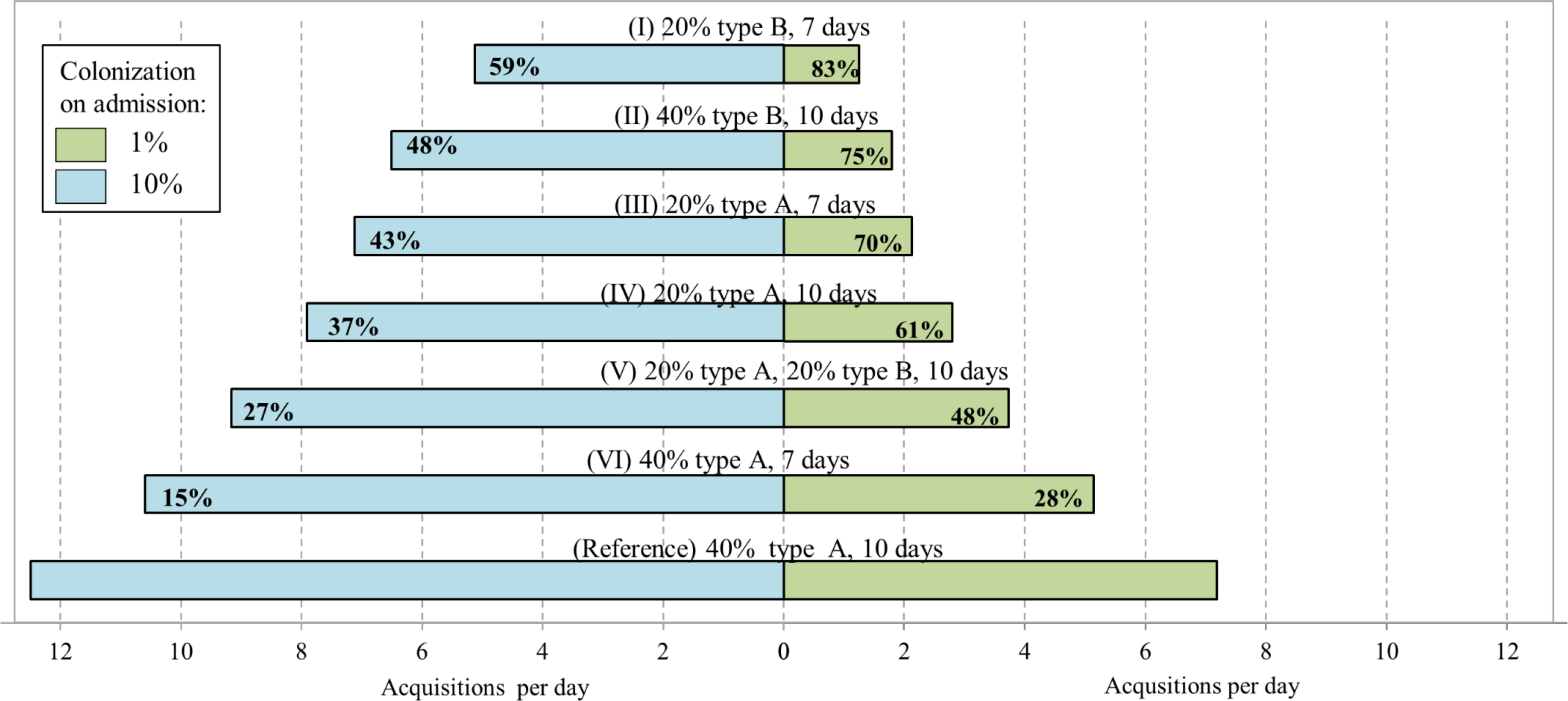
The effect of various antibiotic stewardship interventions on the daily number of ARB-H acquisitions. Prevalence of colonization among admitted patients is 1% and 10%. The note above each bar describes a scenario; % refers to the proportion of admitted patients treated with antibiotics; type is the antibiotic agent, where the characteristics of antibiotic type A are C = 4, V = 4 and of antibiotic type B are C = 2 and V = 2; days refer to duration of treatment. Percentage inside the bar indicates reduction in acquisition relative to the reference scenario.

As presented in Fig 4, stewardship interventions can significantly decrease transmission. The relative impact of the intervention on ARB transmission is higher at an early stage of ARB spread, when 1% of admitted patients are carriers, than when ARB spread is established and 10% are carriers. In the most comprehensive intervention scenario, when the proportion of admitted patients who receive antibiotics is reduced from 40% to 20%, the duration of treatment is reduced from 10 to 7 days, and an antibiotic agent with a lower impact is used (scenario I), acquisitions decreased by 83% and 59%, depending on the prevalence of colonization on admission. In scenario VI, merely shortening the length of treatment from 10 to 7 days reduced acquisitions by 28%. It is also striking that by switching to an antibiotic agent with a lower impact (antibiotic type B) acquisitions were reduced by 75% and 48%, for prevalence of 1% and 10% carriage on admission, respectively (scenario II).

Our results are based on a hospital with characteristics as specified in Table 1. In order to examine how variation in hospital characteristics impacts the ARB-H acquisition rate, we conducted a sensitivity analysis for the nurse-patient ratio, nurses' compliance with infection control measures, and number of patients per ward (S1 Fig). Nurses' compliance has the strongest impact on the rate of ARB-H acquisition: reducing compliance from 50% to 40% more than triples acquisitions, while increasing compliance from 50% to 60% reduces acquisitions by more than half. Reducing the number of patients per ward from 40 to 8 decreases ARB-H acquisitions by 15% -20%. Varying the nurse-patient ratio does not significantly impact the acquisition rate. In all these scenarios, the relative impact of increased antibiotic density on the ARB-H acquisition rate remains similar.

## Discussion

Transmission is the main driver for the spread of most ARB. Controlled experiments to study the effect of antibiotic interventions to reduce transmission of ARB at the hospital level are difficult to perform because of the need to control all potential confounders. This is especially challenging because antibiotic interventions are often implemented as part of a bundle of infection control measures that affect ARB transmission [49].

Therefore, we used ABM to understand the effect of antibiotics on the transmission dynamics and the potential of stewardship interventions. We demonstrated that an increase in antibiotic use increases the incidence of ARB acquisition. At the individual level, the patient's risk increases in parallel with antibiotic density, not only for patients treated with antibiotics but also for those not treated. These effects translate into an exponential increase in incidence of ARB acquisitions when antibiotic density increases and when the antibiotic agent has a high affect.

We found that characteristics of the antibiotic agent used, i.e., its effect on contagiousness and vulnerability, are crucial in determining the antibiotic's impact on the spread of resistance. Our models showed that choosing an agent with a favorable profile in terms of V and C is an important antibiotic stewardship intervention. Yet, in the real world these effects are understudied and the values of V and C for different antibiotic agents are mostly unknown. Moreover, it is very likely that each antibiotic agent has different effects for different ARB. Methods to measure the effects of C and V of antibiotics should be developed and standardized and should be incorporated into drug development programs and drug approval packages.

Our simulations revealed that antibiotic density affects ARB transmission more considerably in the early stages of antibiotic resistance spread, when prevalence among admitted patients is low. Antibiotics have a greater impact on the incidence of ARB acquisition when the ARB strain is highly transmissible. Since highly transmissible strains pose a greater risk to become widespread, antibiotic stewardship interventions can be a powerful tool to combat their spread. We found that reducing antibiotic density has a greater impact on the incidence of ARB acquisition in settings of high antibiotic use. This is an important finding, as variability in antibiotic use between similar wards suggests that reduction in antibiotic density can be achieved in high use settings; such reductions will result in a highly beneficial effect on acquisition of ARB.

Antibiotic use can be viewed as "tragedy of the commons" where individuals, for their own good, exploit a shared limited resource, eventually leading to its depletion [50, 51]. Antibiotic treatment is sometimes portrayed as beneficial at the individual patient level and harmful at the population level [52]. In contrast to this belief, our simulations reveal that the risk of ARB acquisition for a patient treated with antibiotic is substantial in high antibiotic density environments. Clinicians need to recognize this risk when considering antibiotic therapy for their patients in view of the fact that studies suggest that colonization will progress to clinical infection in 8%- 30% of patients colonized by ARB [53–55].

Our study has several limitations. First, we did not take into account the effect of antibiotics on *de novo* emergence of resistance. Second, for simplicity in our scenarios, all antibiotics used in the hospital have uniform characteristics. In the real world a mixture of agents are used simultaneously. This should not change the conclusions derived from our results but will make translation into the hospital setting more complex. Third, we modeled a typical hospital with certain transmission and infection control characteristics. The sensitivity analysis we conducted indicates that the level of staff member compliance with infection control has a significant impact on the rate of acquisitions but the relative effects of antibiotics on the spread of ARB are consistent.

In conclusion, our simulations demonstrate that antibiotic treatment in the hospital setting plays an important role in determining the spread of resistance. It shows that interventions to limit antibiotic use have the potential to rapidly reduce the spread of resistance, and that the risk to the individual patient treated with antibiotics of acquiring antibiotic-resistant bacteria is substantial.

## Supporting information

S1 Fig Sensitivity analysis of the impact of antibiotic density on acquisition of ARB under various hospital characteristics. ARB acquisitions per 100,000 patient days (PD) as antibiotic density increases. (A) The level of nurses' compliance with infection control is: 40%, 50% and 60%; (B) Number of patients per ward is: 8, 16 and 40; (C) Nurse-patient ratio is: 1:8, 1:4, 1:2. In all simulations the resistant bacteria is ARB-H, the characteristics of the antibiotic agent are V = 2 and C = 2, and prevalence of colonization among admitted patients is 1%.
(DOCX)
