# Supplementary material for: The impact of antibiotic use on transmission of resistant bacteria in hospitals: Insights from an agent-based model
Source: PLoS One. 2018 May 14;13(5):e0197111. doi: 10.1371/journal.pone.0197111 (PMC5951570; doi:10.1371/journal.pone.0197111)
Supplement: S1 Fig — ARB acquisitions per 100,000 patient days (PD) as antibiotic density increases. (A) The level of nurses' compliance with infection control is: 40%, 50% and 60%; (B) Number of patients per ward is: 8, 16 and 40; (C) Nurse-patient ratio is: 1:8, 1:4, 1:2. In all simulations the resistant bacteria is ARB-H, the characteristics of the antibiotic agent are V = 2 and C = 2, and prevalence of colonization among admitted patients is 1%. (DOCX) [file pone.0197111.s001.docx]

**Supporting information**

In the simulations presented in the paper we used a hospital where: the nurse-patient ratio is 1:8, nurses comply with infection control measures in 50% of opportunities, and each ward includes 40 patients.

We conducted a sensitivity analysis in order to examine how variation in these parameters impacts ARB acquisition dynamics. In the simulations the resistant bacteria is ARB-H, the characteristics of the antibiotic agent are V = 2 and C = 2, and prevalence of colonization among admitted patients is 1%. In order to examine one hospital characteristic at a time, when varying the number of patients in a ward we maintain the nurse-patient ratio of 1:8. When varying the nurse-patient ratio we maintain the average number of contacts a patient has with a nurse equal to 12 per day.

Nurses' compliance with infection control measures has the strongest impact on the rate of ARB acquisition (Fig S1A). Reducing compliance from 50% to 40% results in more than 3 times more ARB acquisitions, while increasing compliance from 50% to 60% reduces ARB-H acquisitions by more than half.

The impact of the two other characteristics is not as strong. Reducing the number of patients per ward from 40 to 8 decreases ARB-H acquisitions by 15% -20%, depending on antibiotic density (Fig. S1B). The variations in the ratio of nurse-patient does not significantly impact the acquisition rate (Fig. S1C).

While the number of ARB-H acquisition varies among the tested characteristics the relative impact of increased antibiotic density on ARB-H acquisition rate remains similar.

In reality, the three hospital characteristics that we examined may not be independent as in the simulation. Decreasing the number of patients per nurse would reduce the workload, and thus compliance with infection control measures may increase. When nurses are assigned to fewer patients, it is also likely that the number of contacts between a patient and nurse will increase. The results of our sensitivity analysis should be considered in light of the dependency between these parameters.


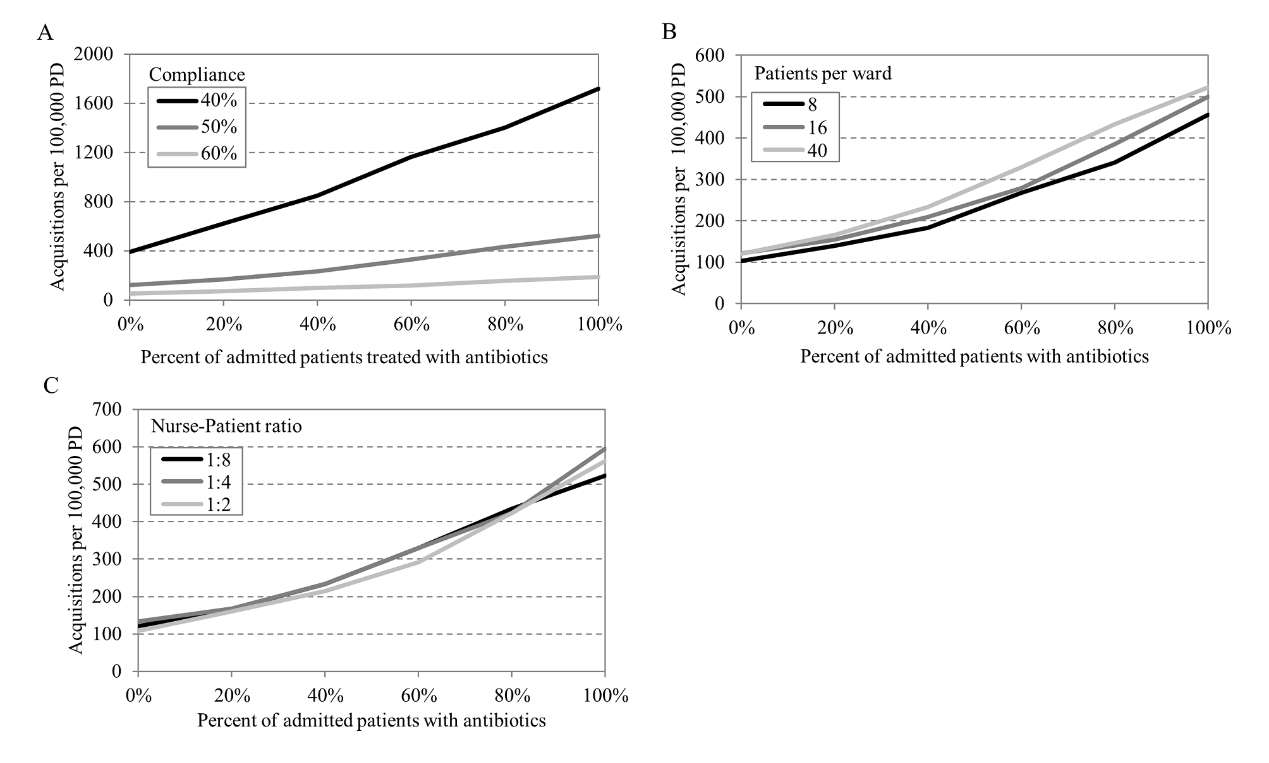


**S1 Fig. Sensitivity analysis of the impact of antibiotic density on acquisition of ARB under various hospital characteristics.** ARB acquisitions per 100,000 patient days (PD) as antibiotic density increases. (A) The level of nurses' compliance with infection control is: 40%, 50% and 60%; (B) Number of patients per ward is: 8, 16 and 40; (C) Nurse-patient ratio is: 1:8, 1:4, 1:2. In all simulations the resistant bacteria is ARB-H, the characteristics of the antibiotic agent are V = 2 and C = 2, and prevalence of colonization among admitted patients is 1%.
